# An Attention-Sensitive Memory Trace in Macaque MT Following Saccadic Eye Movements

**DOI:** 10.1371/journal.pbio.1002390

**Published:** 2016-02-22

**Authors:** Tao Yao, Stefan Treue, B. Suresh Krishna

**Affiliations:** 1 Cognitive Neuroscience Laboratory, German Primate Center, Goettingen, Germany; 2 Bernstein Center for Computational Neuroscience, Goettingen, Germany; 3 Faculty of Biology and Psychology, Goettingen University, Goettingen, Germany; McGill University, CANADA

## Abstract

We experience a visually stable world despite frequent retinal image displacements induced by eye, head, and body movements. The neural mechanisms underlying this remain unclear. One mechanism that may contribute is transsaccadic remapping, in which the responses of some neurons in various attentional, oculomotor, and visual brain areas appear to anticipate the consequences of saccades. The functional role of transsaccadic remapping is actively debated, and many of its key properties remain unknown. Here, recording from two monkeys trained to make a saccade while directing attention to one of two spatial locations, we show that neurons in the middle temporal area (MT), a key locus in the motion-processing pathway of humans and macaques, show a form of transsaccadic remapping called a memory trace. The memory trace in MT neurons is enhanced by the allocation of top-down spatial attention. Our data provide the first demonstration, to our knowledge, of the influence of top-down attention on the memory trace anywhere in the brain. We find evidence only for a small and transient effect of motion direction on the memory trace (and in only one of two monkeys), arguing against a role for MT in the theoretically critical yet empirically contentious phenomenon of spatiotopic feature-comparison and adaptation transfer across saccades. Our data support the hypothesis that transsaccadic remapping represents the shift of attentional pointers in a retinotopic map, so that relevant locations can be tracked and rapidly processed across saccades. Our results resolve important issues concerning the perisaccadic representation of visual stimuli in the dorsal stream and demonstrate a significant role for top-down attention in modulating this representation.

## Abstract 2

How does the brain keep track of specific attended features after eye movements? A new study of the macaque brain implicates the middle temporal (MT) area in the remapping of attentional pointers across saccades.

## Author Summary

Humans experience a visually stable world despite the fact that eye, head, and body movements cause frequent shifts of the image on the retina. Humans and monkeys are also able to keep track of visual stimuli across such movements. One mechanism that may contribute to these abilities is “transsaccadic remapping,” in which the responses of some neurons in various attentional, oculomotor, and visual brain areas appear to anticipate the consequences of saccades. A current hypothesis proposes that the brain maintains “attentional pointers” to the locations of relevant stimuli and that, via transsaccadic remapping, it rapidly relocates these pointers to compensate for intervening eye movements. Whether stimulus features are also remapped across saccades (along with their location) remains unclear. Here, we show the presence of transsaccadic remapping in a macaque monkey brain area critical for visual motion processing, the middle temporal area (MT). This remapped response is stronger for an attended stimulus. We find only weak evidence for motion-direction information in the remapped response. These results support the attentional pointer hypothesis and demonstrate for the first time, to our knowledge, the impact of top-down attention on transsaccadic remapping in the brain.

## Introduction

Prior research has revealed the potential contribution to visual processing of transsaccadic remapping, in which some neurons in the lateral intraparietal area (LIP), frontal eye field (FEF), superior colliculus (SC), medial superior temporal area (MST), and ventral stream (areas V3a, V3, and V2) respond perisaccadically as long as a visual stimulus could be anticipated in their receptive fields (RFs) after the saccade [1–7]. This “remapped response” is not a simple visual afferent response, because it appears even when the visual stimulus disappears just before the saccade (that would bring the stimulus location into the RF), so that no stimulus ever appears in the neurons’ visual RF before or after the saccade. Furthermore, in some neurons, it begins with a latency shorter than the normal visual latency and can even begin before saccade onset, in which case it has been referred to as “predictive remapping” [1]. More commonly, the remapped response occurs postsaccadically, and when this occurs in a situation in which there is no postsaccadic stimulus in the RF because it disappeared before the saccade, the remapped response is referred to as a “memory trace” of the location of the visual stimulus [1].

The functional role of this remapped response is currently being actively debated, and many of its key response properties remain unknown. It has been proposed [8] that transsaccadic remapping represents the predictive, presaccadic shift of attentional pointers on a retinotopic map that keeps track of attended locations across saccades, so that attended locations can be preferentially processed with minimal delay after the saccade. This reduction of delay would be especially helpful when planning rapid sequential saccades and could also help maintain an uninterrupted visual experience across saccades. Others have suggested that this view may be too restrictive and that information about visual features are also remapped across saccades, in addition to location [9–12]. This alternative view thus invokes an additional role for transsaccadic remapping in spatiotopic feature comparison and adaptation transfer across saccades. Resolving these issues requires a better understanding of the properties of the remapped response in different brain areas. Here, we address and answer several open questions regarding the remapped response in the middle temporal area (MT), a key motion processing area, in the rhesus macaque.

MT is an important locus in the processing of visual motion and is strongly interconnected with LIP, FEF, SC, and MST. A previous report from macaque MT showed the absence of predictive remapping [13] in MT neurons; our results are consistent with this. Another recent report reported the presence of a memory trace in MST neurons but failed to find a memory trace in a small sample of MT neurons using a paradigm with a flashed visual stimulus; the authors therefore suggested that the memory trace may be an emergent property that differentiates MST from MT [2]. Here, we report that MT neurons do show a memory trace, using an experimental paradigm that requires the monkey to pay top-down attention to one of two motion stimuli. Furthermore, we show that the memory trace in MT neurons is enhanced by top-down spatial attention. This is the first demonstration, to our knowledge, of the influence of top-down attention on the memory trace in any brain area. Finally, we find evidence only for a weak and transient effect of motion direction on the memory trace in one monkey. Our data are therefore consistent with the hypothesis that transsaccadic remapping represents the shift of attentional pointers in a retinotopic map. Our results further clarify the perisaccadic representation of visual stimuli in the dorsal stream and demonstrate a significant role for top-down attention in modulating this representation.

## Results

**Fig 1:**
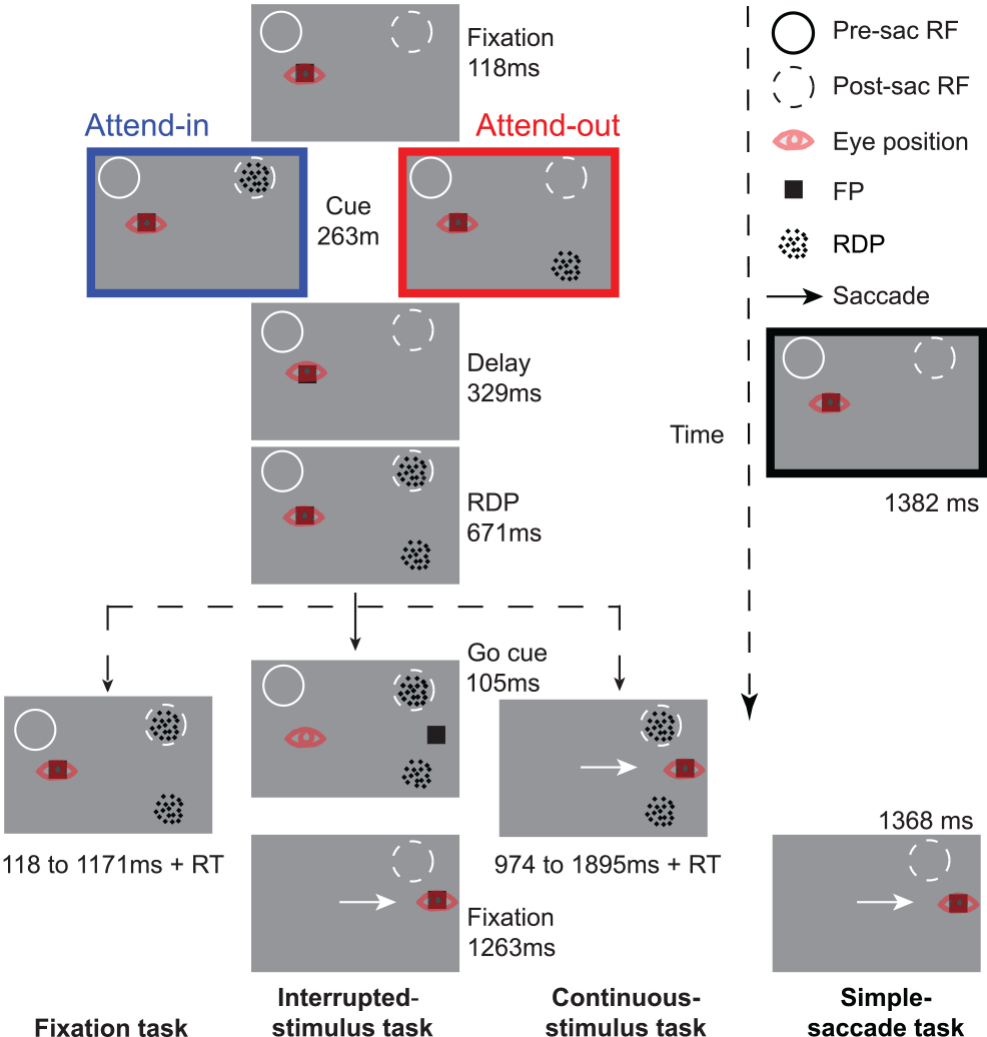
Task design and timing. Two rhesus monkeys were trained to perform a task that involved attending to one of two moving RDPs while also making a visually guided saccade if the fixation point jumped to a new location (continuous-stimulus task and fixation task). On about 44% of trials, the RDPs disappeared just before the saccade (interrupted-stimulus task). On about 11% of trials, RDPs were never presented and the monkey only had to make a visually guided saccade (simple-saccade task). Values next to each panel represent the durations of the task phase represented by that panel. For details, see Materials and Methods and S1 Text.

**Fig 2:**
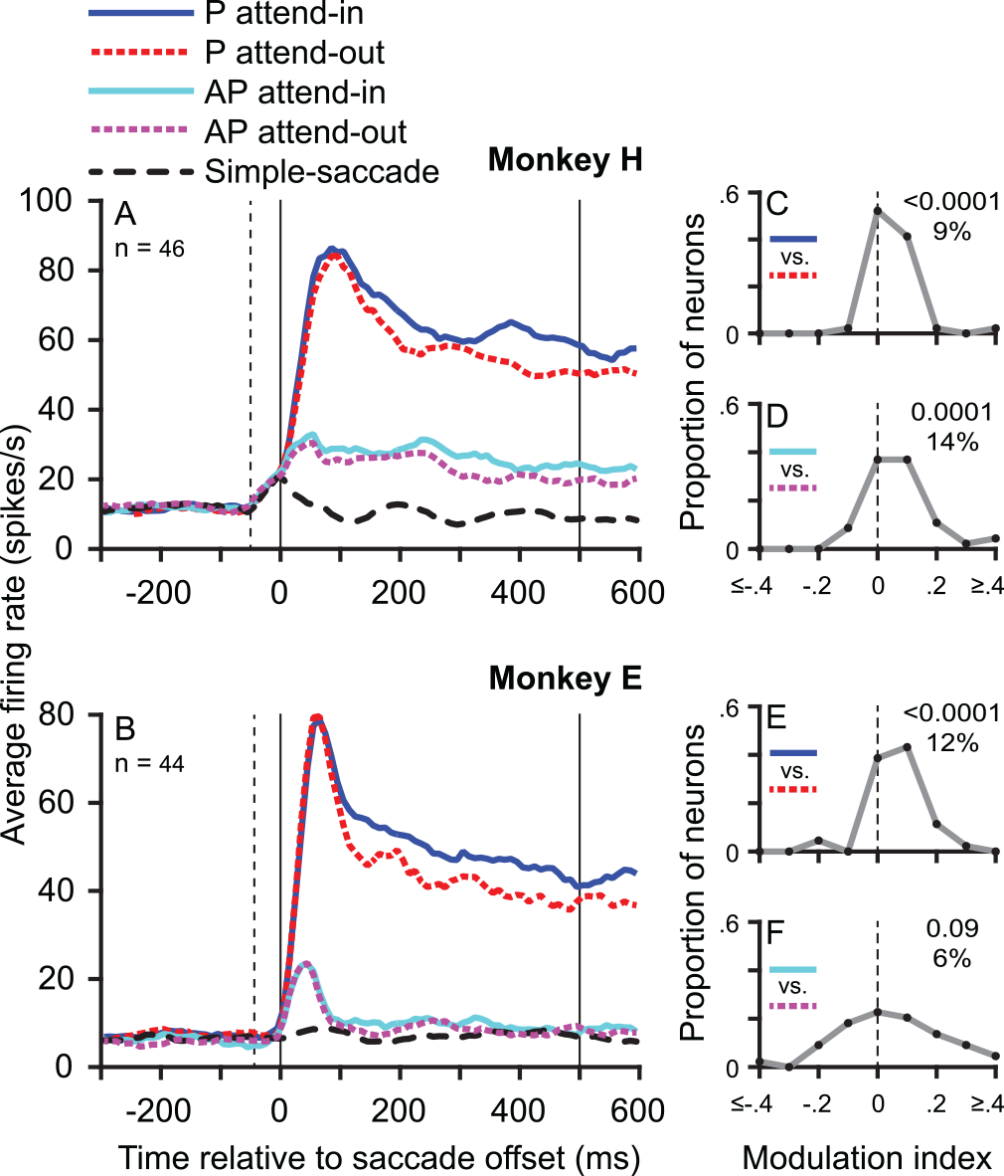
Attentional enhancement of MT neuronal responses to moving RDPs. (A,B) Population average peristimulus time histograms (PSTHs) for monkey H (A) and monkey E (B) in response to attended (target) and unattended (distractor) RDPs moving either in the preferred (P) or antipreferred (AP) direction (see legend at top left). The PSTH for the simple-saccade task is also shown in black as a reference. Solid vertical lines demarcate the time window used for computing the modulation indices in C–F. The dotted vertical line indicates the mean time of saccade onset. (C,E) Frequency polygons of the distribution of modulation indices in monkey H (C) and monkey E (E) when the preferred direction RDP is in the RF after the saccade show a clear predominance of values greater than zero, i.e., an enhanced response in the attend-in conditions. The *p*-value from the signed-rank test of the modulation indices and the median modulation index (converted to a percentage and rounded) are shown on the top right of each panel. The final point in the frequency polygons sums all data values at or beyond the extreme value. (D,F) Same as C,E but for the antipreferred direction RDP. Data in Supporting Information (S1 Data).

We report the responses of 90 MT neurons, 46 from monkey H and 44 from monkey E. We first considered the responses from 0 to 500 ms after saccade offset (see Methods) in the continuous-stimulus task (Fig 1), in which the monkeys made a saccade that brought either the (previously cued) target random dot pattern (RDP) or the distractor RDP into their RF, and no stimulus was present in the RF before the saccade. As previously reported (e.g., [14–16]), neurons showed a clear postsaccadic enhancement for attended (solid blue curves, Fig 2A and 2B) versus unattended (dotted red curves, Fig 2A and 2B) RDPs moving in the preferred direction in their RF. Compared to the distractor RDP, the response to the (cued) target RDP moving in the preferred direction in a time window of 0 to 500 ms following saccade offset was greater by a median value of 8.9% in monkey H (Fig 2C, *p* < 0.0001) and 12.4% in monkey E (Fig 2E, *p* < 0.0001). The attentional modulation of an antipreferred direction target was significant in monkey H (Fig 2D, 13.7%, *p* = 0.0001) but not in monkey E (Fig 2F, 5.8%, *p* = 0.0909). Consistent with a prior report [13], we observed no predictive remapping, i.e., no presaccadic increase in activity in MT neuronal responses (Figs 2A, 2B and S1).

### MT Neurons Show a Memory Trace That Is Enhanced by Top-Down Attention

**Fig 3:**
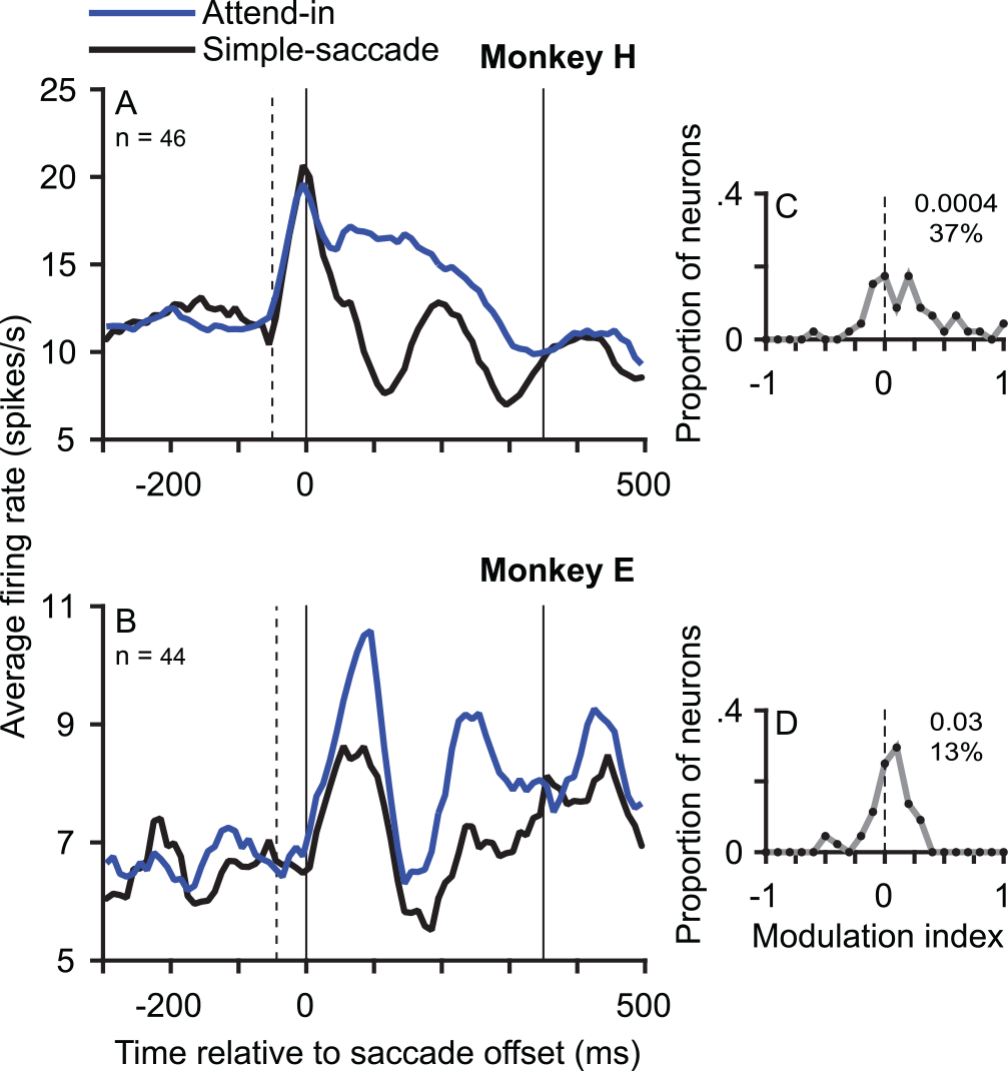
MT neurons show a memory trace. The memory trace is an enhanced postsaccadic response (compared to the simple-saccade) when a moving target RDP was presented (only before the saccade) at their postsaccadic RF location. We pooled the responses to the two RDP directions because we did not find an influence of RDP motion direction on the memory trace (Fig 5). (A,B) Population average PSTHs for monkey H (A) and monkey E (B) in the attend-in condition of the interrupted-stimulus task (blue) compared to the simple-saccade task (black). The *y*-axes in A and B have different ranges. (C,D) Frequency polygons of the distribution of modulation indices (for the response from 0 to 350 ms after saccade offset) comparing these two conditions in monkey H (C) and monkey E (D) show a clear predominance of values greater than zero, i.e., an enhanced response in the attend-in condition. Conventions as in Fig 2. Data in Supporting Information (S2 Data).

In contrast to the continuous-stimulus task above, the RDPs in the interrupted-stimulus task (Fig 1) disappear before saccade onset. Thus, a neuronal response after the saccade would represent a memory trace and not a sensory response, since there is no stimulus in the postsaccadic RF (or in the presaccadic RF). To determine the presence of a memory trace, we considered the responses in the interrupted-stimulus task when the target RDP (irrespective of motion direction) was at the postsaccadic RF location before the saccade (attend-in interrupted-stimulus condition). We compared this response from 0 to 350 ms after saccade offset (see Methods) to the response in the same time window in the simple-saccade task where the monkey only made a saccade with no RDP ever appearing on the screen (Fig 3). We found a strong enhancement of responses in the attend-in condition of the interrupted-stimulus task compared to the simple-saccade task (Fig 3A and 3B, blue curve versus black curve), and we interpret this enhancement as a memory trace of the visual stimulus presented before the saccade. The median enhancement of the response following saccade offset in the attend-in interrupted-stimulus condition was 37.4% (*p* = 0.0004) in monkey H and 13.1% (*p* = 0.0308) in monkey E.

**Fig 4:**
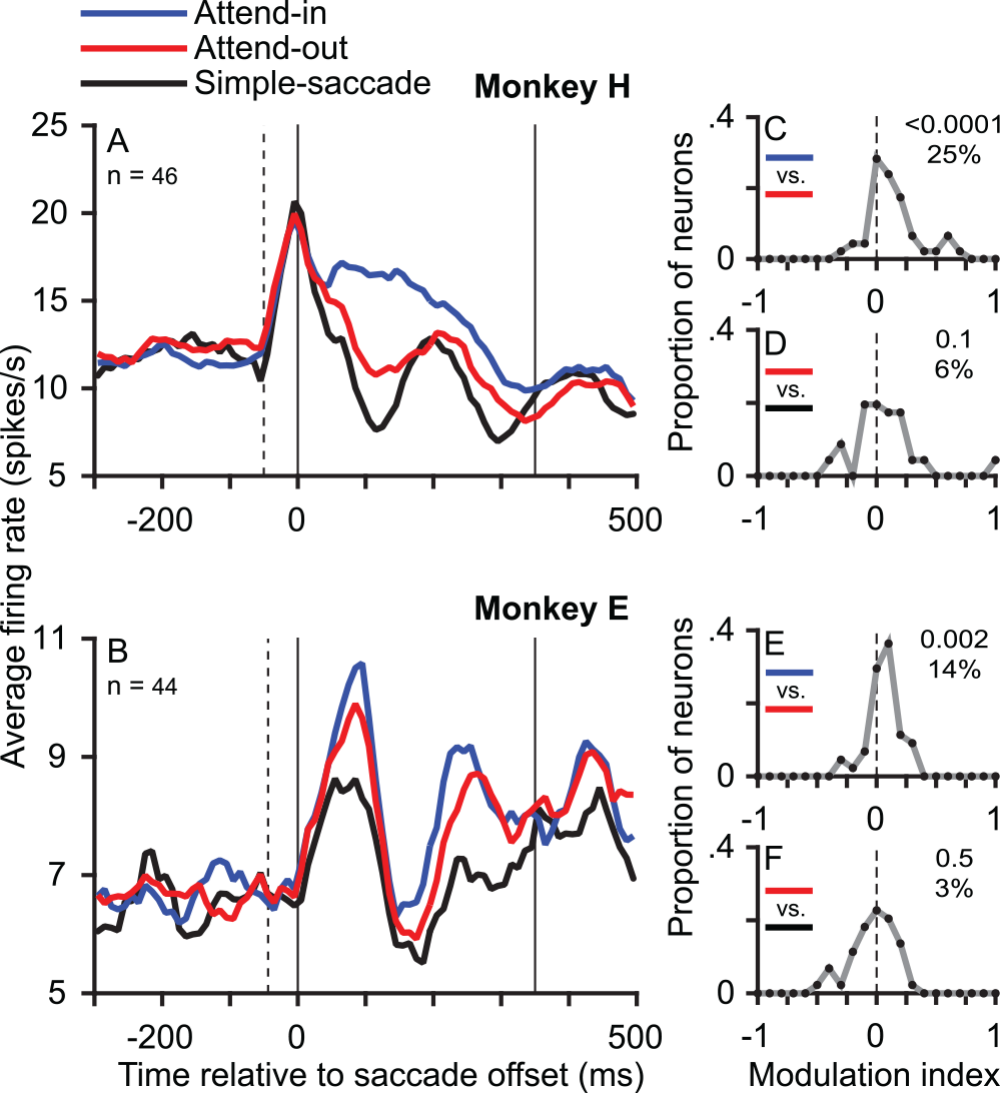
The memory trace is sensitive to top-down attention. The postsaccadic response is larger when a target RDP (as compared to a distractor RDP) was in the postsaccadic RF location before the saccade. (A,B) Population average PSTHs for monkey H (A) and monkey E (B) in the attend-in condition (blue) and attend-out condition (red) of the interrupted-stimulus task, pooled across motion directions as well as the simple-saccade task (black). The *y*-axes in A and B have different ranges. (C,E) Frequency polygons of the distribution of modulation indices comparing the attend-in and attend-out conditions in monkey H (C) and monkey E (E) show a clear predominance of values greater than zero, i.e., an enhanced response in the attend-in condition. (D,F) Frequency polygons of the distribution of modulation indices comparing the attend-out condition of the interrupted-stimulus task and the simple-saccade task in monkey H (D) and monkey E (F) show no significant difference in the responses. Conventions as in Figs 2 and 3. Data in Supporting Information (S3 Data).

In order to examine the effect of attention on the memory trace, we then compared the memory trace for the target (in the attend-in interrupted-stimulus condition) to that for the distractor RDP (in the attend-out interrupted-stimulus condition): in both cases, the RDP was in the postsaccadic RF before the saccade but not after it. The memory trace for the target was clearly greater than that for the distractor RDP (Fig 4A and 4B, blue curve versus red curve). The median enhancement of the memory trace for the target relative to that for the distractor was 25.4% (*p* < 0.0001, Fig 4C) in monkey H and 14.1% (*p* = 0.0022, Fig 4E) in monkey E. The memory trace for the distractor, on the other hand, was either weak or absent. Based on the modulation indices, the memory trace for the distractor was not significantly different from when there was no stimulus in the simple-saccade condition (Fig 4A and 4B, red curve compared to black curve): the response in the attend-out condition was larger by 6.4% (*p* = 0.1417, Fig 4D) in monkey H and by 2.7% (*p* = 0.5222, Fig 4F) in monkey E. However, this lack of significance appears to contrast with the effect that is visible in the average population PSTHs (red versus black curves in Fig 4A and 4B). This is because the separation between the average population PSTHs reflects the difference between the mean firing rates in the two conditions, while the median modulation index is a measure based on the ratio of firing rates. Performing a paired *t* test between the firing rates in the attend-out and simple-saccade condition does reveal a significant enhancement in the attend-out condition (Monkey H: mean difference = 1.3 spikes per second, *p* = 0.0450; Monkey E: mean difference = 0.8 spikes per second, *p* = 0.0270).

### The MT Memory Trace Only Shows a Transient Effect of Motion Direction (In One Monkey)

**Fig 5:**
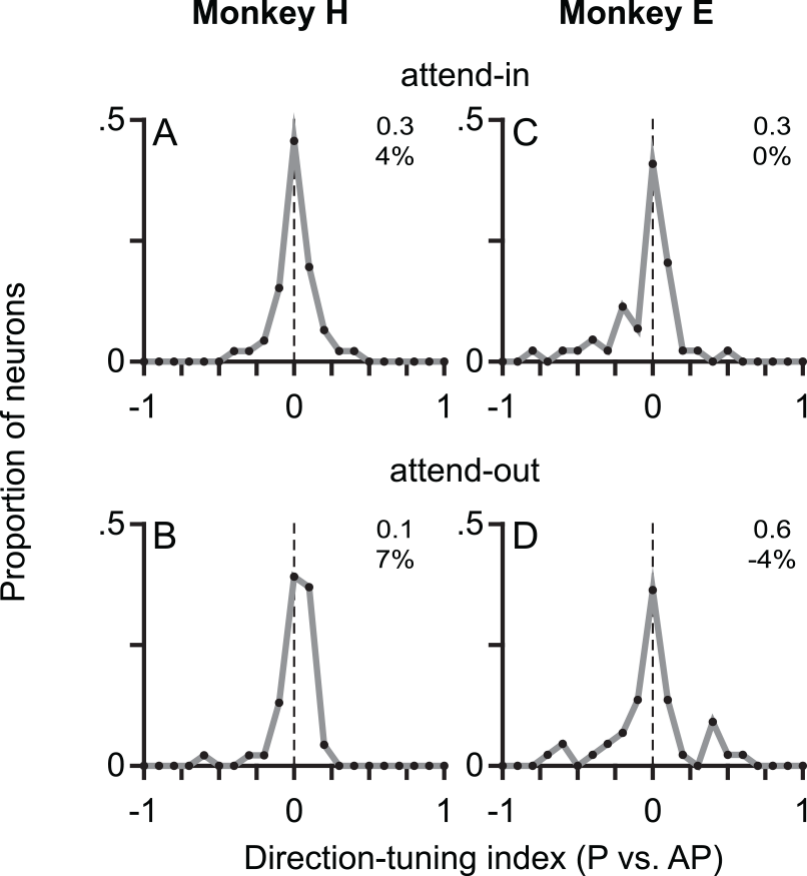
The memory trace (from 0 to 350 ms) is not sensitive to motion direction. The postsaccadic response is not significantly different when the preferred (P) or antipreferred (AP) direction RDP was in the postsaccadic RF location before the saccade. The four panels show frequency polygons of the distribution of direction-tuning indices (see Methods) for the attend-in (A,C) and attend-out (B,D) conditions of the interrupted-stimulus task (for the response from 0 to 350 ms after saccade offset). Results for monkey H in the left column (A,B) and for monkey E in the right column (C,D). None of the distributions show a statistically significant deviation from zero. Preferred and antipreferred directions were defined based on the response in the continuous-stimulus task. Other conventions as in Figs 2–4. Data in Supporting Information (S4 Data).

We examined whether the attention-sensitive memory trace in MT also contains information about the motion direction of the stimulus that elicited the memory trace, whether it be the target or the distractor RDP. We calculated the responses for trials in which the preferred or antipreferred direction RDP (as identified from the continuous-stimulus task) was in the postsaccadic RF. We did not find any significant effect of motion direction when we compared the responses in either the attend-in condition (with the target RDP in the postsaccadic RF location) or the attend-out condition (with the distractor RDP in the postsaccadic RF location). None of the response differences (Fig 5A–5D) were statistically significant (all *p*-values > 0.1248). Additionally, since the preferred and antipreferred directions defined on the basis of the responses in the continuous-stimulus task may not predict the memory trace in the interrupted-stimulus task, we used a two-fold approach. We first computed the response after saccade offset on even-numbered trials and designated the motion direction that elicited the larger response as the preferred direction. We then used odd-numbered trials to perform the same analysis of the effects of motion direction on the memory trace. Once again, none of the response differences were statistically significant (all *p*-values > 0.1292).

**Fig 6:**
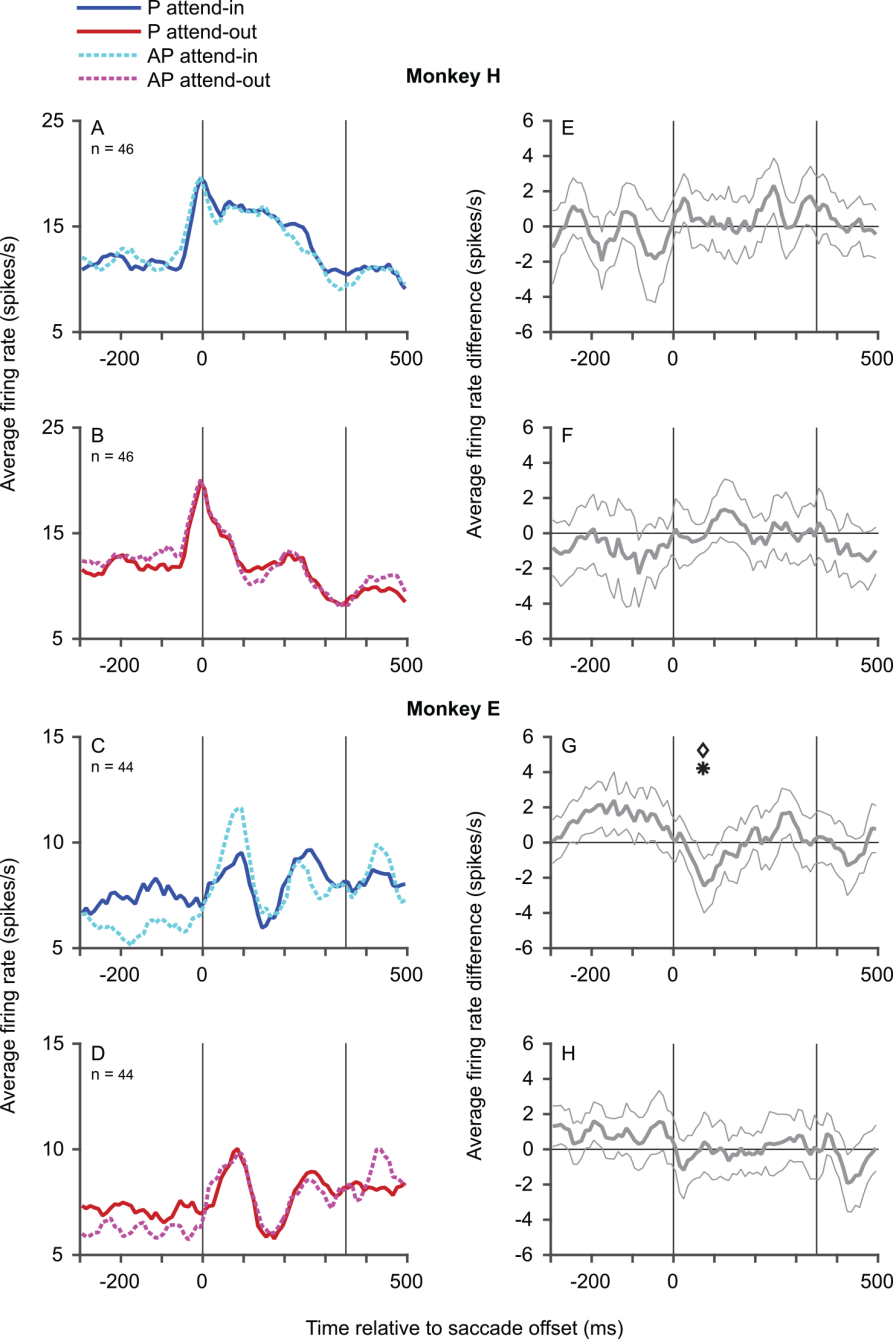
Evidence only for transient feature-related information in the memory trace in one monkey. In the left column, population average PSTHs for the preferred direction (blue trace in A,C and red trace in B,D) and nonpreferred direction (cyan trace in A,C and magenta trace in B,D) for the attend-in (A,C) and attend-out (B,D) conditions of the interrupted-stimulus task are plotted. Preferred and nonpreferred directions were determined from the responses in the continuous-stimulus task. In the right column, the mean difference (averaged across neurons) between the PSTH for the preferred and nonpreferred direction is plotted (along with the 95% confidence bands) for the attend-in (E,G) and attend-out (F,H) conditions. All PSTHs calculated using overlapping 50 ms bins, stepping every 10 ms. Data from monkey H (A–B,E–F) and monkey E (C–D,G–H). Statistical significance was calculated (using signed-rank tests Bonferroni-corrected for 16 comparisons) using the difference between firing rates (diamond symbols) as well as using the modulation index (asterisk symbol) in nonoverlapping 50 ms windows over the entire analysis period (16 comparisons over 800 ms from -300 to 500 ms relative to saccade offset). Only one time bin (G) showed significance. Other conventions as in Fig 2. Data in Supporting Information (S5 Data).

Since it is possible that motion-direction selectivity may be present in the memory trace at shorter time scales, we also examined the motion-direction selectivity of the memory trace over shorter time periods (Fig 6). There was no evidence for motion-direction selectivity in the memory trace for monkey H in either the attend-in (Fig 6A and 6E) or the attend-out (Fig 6B and 6F) conditions, as evidenced by the fact that the 95% confidence bands (Fig 6E and 6F) included zero throughout the time course and none of the nonoverlapping statistical comparisons (Bonferroni-corrected for multiple comparisons) were statistically significant. The results from monkey E were similar, except that there was a transient effect of motion direction on the memory trace (Fig 6C and 6G) in the attend-in condition, where the memory trace for the nonpreferred direction was larger in the time window from 50 to 100 ms after saccade offset (*p* = 0.0031 for the direction-tuning index and 0.0024 for the difference in firing rates). Examining the response in this time window in each individual neuron (using a rank-sum test comparing the responses to the preferred and nonpreferred directions) did not yield significance for any neuron. Also, using a more liberal false-discovery rate correction for multiple comparisons did not change the result for the post-saccadic time bins, but indicated a significant effect of motion direction before the saccade (as suggested by the PSTHs in Fig 6C, with the responses in the blue trace being slightly larger than the cyan trace). These presaccadic effects could reflect weak stimulus-driven effects from outside the RF [17] and/or the effects of feature-based attention [18].

In both monkeys, a transient increase in activity that starts either before or immediately after saccade offset is visible in the average population PSTHs from all three tasks: the simple-saccade task, the interrupted-stimulus task (Fig 4A and 4B), and the continuous-stimulus task (Fig 2A and 2B). For the simple-saccade task, similar responses have been reported before, with substantial variability in individual neurons ([19,20]; also see Discussion). The apparent difference in time course in this response between the two monkeys may represent differences in the sampled population of neurons, since a subset of neurons (with more eccentric RFs) from monkey E shows a time course quite similar to that in monkey H. The genesis and properties of this response difference, though not fully understood, are beyond the focus of this paper, since our experiment was not designed to study it.

## Discussion

We report that MT neurons show a memory trace: they respond more strongly after a saccade when a stimulus is present only before the saccade in their postsaccadic RF location. Furthermore, we show that the memory trace is stronger for an attended stimulus and does not contain information about motion direction. A memory trace has been shown previously in areas like LIP, FEF, SC, and MST [1–3,5,6], with which MT is strongly anatomically connected [21–23]. Another recent study by Inaba and Kawano [2] reported that a sample of 46 MT neurons did not carry information about the location of a recently disappeared stimulus in their postsaccadic response; they only found such information in the responses of MST neurons and concluded that the memory trace was an emergent property of MST neurons. Our task differs from theirs because it required the monkey to pay (top-down) attention to one of two stimuli, while their task only required the monkey to make a simple, visually guided saccade and the stimulus used to probe the memory trace was task-irrelevant. The difference between our results might therefore be partially explained by our result that MT neurons show a clear memory trace for attended stimuli, with the memory trace for unattended stimuli being weak or absent. However, other aspects might also contribute. First, the two studies probably sampled different kinds of neurons: the study by Inaba and Kawano excluded neurons that showed a response in the simple-saccade task, while the neurons in our sample show a transient response in the simple-saccade task (Fig 3). Such a transient response has been reported before from area MST [19] as well as area MT, even following saccades made in the dark (Ibbotson, M.R., personal communication, even though an earlier report from Ibbotson and colleagues [24] reported the absence of such a response in a small sample of 17 MT neurons). Further supporting the possibility that different neurons were sampled, the study by Inaba and Kawano reported that MT neurons showed a substantially longer latency (relative to saccade offset) to stimuli brought into their RFs by a saccade compared to stimuli flashed in their RF. This differs from the conclusion reached by another recent study [13] in which no difference was found between the two latencies. Though we do not present an analysis of response latency here, the average population PSTHs (Fig 2A and 2B) suggest that the average latency is not longer than that expected from previous reports on MT neurons (between 30 and 40 ms [2,13]). Second, the polarity of the stimuli used was also different: the study by Inaba and Kawano used a white stimulus on a dark background, while we used a dark stimulus on a white background, and such polarity differences are known to have strong effects on the responses of V1 neurons [25]. Finally, the study by Inaba and Kawano relied on a receiver operating characteristic analysis performed within a sliding 10 ms window to report the absence of spatial tuning in the postsaccadic response 0 to 100 ms after saccade offset. Based on our results, it is possible that the 10 ms window may be too narrow and the 100 ms window too short to reliably detect tuning.

We show that the memory trace in MT neurons is larger for an attended stimulus; this is the first demonstration of the influence of top-down attention on the memory trace in any brain area. The task required the monkey to attend to the stimulus location throughout the trial. This stimulus location lay outside the recorded neuron’s RF before the saccade and inside it only after the saccade. Therefore, assuming a single locus of attention, the monkey would have to shift attention from its presaccadic location on a retinotopic map (outside the RF) to its postsaccadic location (inside the RF) right around the saccade. It is also possible that two loci of attention simultaneously exist and that, around the saccade, attention is allocated simultaneously to both task-relevant locations (the presaccadic and postsaccadic stimulus locations on the retinotopic map). Previous psychophysical data from humans indicate that attentional effects are visible at the postsaccadic retinotopic location of a task-relevant stimulus shortly before saccade onset [26–28]. In our task, the neural data from MT indicate that attentional effects emerge in MT soon after saccade offset, but not before that (Figs 2 and S2). Based on our results, we suggest that the memory trace can be explained as the postsaccadic enhancing effect of a perisaccadic allocation of attention to the RF location (on a retinotopic map), where the monkey expects the target to be. Since there is no longer a stimulus in the neuron’s RF, attention acts on the baseline, stimulus-independent activity to produce the memory trace. Psychophysically, attentional effects may have been visible in our task either before the saccade (though MT only shows postsaccadic attentional effects) or after the saccade (around the same time as the emergence of attentional effects in MT); since our task design did not allow us to measure the dynamics of attention psychophysically, we cannot distinguish between these two possibilities. Our interpretation of the memory trace as reflecting a top-down attentional effect in our task is consistent with previous findings showing stronger remapped responses for salient or task-relevant stimuli: LIP neurons show stronger levels of anticipatory (predictive) remapping to the appearance of a visual search target [29] or saccade target [30] in their RF after a saccade, compared to the appearance of a distractor. Similarly, stimuli with greater bottom-up saliency have been shown to elicit stronger remapped responses in LIP [30] and FEF [31]. When using a measure based on the difference of spike rates, our data indicate a weak memory trace for the unattended stimulus. However, this effect is not present when using a measure based on the ratio of spike rates. More data with a greater number of stimuli are needed before reaching more general conclusions about the extent to which unattended stimuli are also transsaccadically remapped.

The phenomenology of presaccadic remapping of visual RFs measured using flashed stimuli is currently controversial. The classical position in the literature is that neurons that show predictive remapping in LIP [1], FEF [4], and SC [6] are anticipating the appearance of a stimulus in their postsaccadic RF (as if they are shifting their RFs preemptively to their future postsaccadic locations). In contrast, it has recently been proposed [32,33], based on recordings from FEF, that such remapping actually represents a transient shift of visual RFs toward the saccade target and that this transient shift is correlated with the attentional shift to the saccade target before the saccade [34,35]. While these two views await reconciliation, we emphasize that our experimental design and interpretation of the memory trace in MT is not dependent on either of these competing accounts of the phenomenology of predictive visual RF shifts measured using flashed stimuli. Our design and interpretation instead depend only on the fairly large body of evidence supporting spatially accurate remapping: psychophysical evidence from double-step experiments [36], free-viewing visual search [37], and transsaccadic attentional measurements [15,27] all show that the locations of salient stimuli and future saccade targets are remapped rapidly and accurately across saccades. Similarly, LIP [29,30] and FEF [38] neurons anticipatorily signal the presence of a target in their RFs. SC, LIP, and FEF neurons signal the location of the impending second saccade within their RFs in a double-step task [39–42]. SC neurons also rapidly compensate for midsaccade deviations in eye position introduced by electrical stimulation in the SC during a saccade [43,44]. The relationship of this spatially accurate remapping mechanism to the contentious spatial properties of predictive visual RF shifts is unclear at present.

The presence of feature-related information in the remapped response has become a critical test that distinguishes between two alternative views of the functional role of transsaccadic remapping that are being actively debated [8,9,11,32,45]. Absence of featural information in the remapped response would support the proposal [8] that transsaccadic remapping represents the predictive, presaccadic shift of attentional pointers on a retinotopic map that keeps track of attended locations across saccades. On the other hand, the presence of featural information in the remapped response would support the proposal that transsaccadic remapping plays an additional role in spatiotopic feature comparison and adaptation transfer across saccades [9–12], though the data on adaptation transfer have not been universally replicated (summarized in [46]). Our data clearly indicate that any motion-direction information present in the remapped response is weak: in our data, it was only present transiently in one monkey. This, combined with the greater memory trace elicited by the attended stimulus, indicates that the memory trace in MT neurons predominantly represents the effects of a shift of attentional pointers. We note here that evidence for featural information in the remapped response has been presented recently from LIP [47], and neurons that signal transsaccadic changes in stimulus location and/or color in their postsaccadic reafferent response have been found in FEF (though its relationship to remapping is unclear [12]). However, these areas only show coarse tuning to stimulus features, and our data provide the first set of evidence (against a feature-tuned remapped response) from a sensory area with neurons that are more finely tuned to stimulus features. The weak effect found in our data may either reflect a feature-selective input to the remapped response or simply the effects of response adaptation given the slightly higher response in the presaccadic period when the monkey was attending to the preferred direction outside the RF. These presaccadic effects could themselves reflect weak stimulus-driven effects from outside the RF [17] and/or the effects of feature-based attention [18].

MT neurons do not show anticipatory remapping [13]; we hypothesize that the anticipatory remapping seen in attentional and oculomotor control areas like LIP, FEF, and SC is part of the process that switches the attentional pointer, and though this process starts before the saccade in these areas, its effects in MT only manifest after the saccade (at a point of time when the pointer is again at the task-relevant location). The anticipatory nature of the remapping seen in LIP, FEF, and SC may confer an evolutionary advantage by ensuring that attention is allocated to the correct retinotopic location soon after the saccade. A recent psychophysical study [28], using a motion task similar to ours, observed a decrement in performance at attended locations before a saccade and suggested that this resulted from the known reallocation of attention to the saccade target [34,48–50] and/or the remapped location [27]. Our data from MT do not indicate any evidence for a presaccadic shift of attention to the remapped location. Recordings from areas upstream of MT along the motion-process pathway combined with psychophysical measurements of perisaccadic attentional dynamics are needed before the neural basis of these processes can be understood. Our current results, when combined, resolve important issues concerning the perisaccadic representation of visual stimuli in the dorsal stream and demonstrate a significant role for top-down attention in modulating this representation.

## Materials and Methods

### Statement on Animal Research within This Study

All animal work was conducted according to the relevant national and international guidelines. All animal procedures have been approved by the responsible regional government office (Niedersächsisches Landesamt für Verbraucherschutz und Lebensmittelsicherheit [LAVES]) under the permit numbers 33.14.42502-04-064/07 and 3392 42502-04-13/1100.

The animals were group-housed with other macaque monkeys in facilities of the German Primate Center in Goettingen, Germany in accordance with all applicable German and European regulations. The facility provides the animals with an enriched environment (including a multitude of toys and wooden structures) exceeding the size requirements of the relevant European regulations.

All invasive procedures were done under appropriate anesthesia and with appropriate analgesics. The German Primate Center has several veterinarians on staff that regularly monitor and examine the animals and consult on any procedures.

During the study, the animals had unrestricted access to food and fluid, except on the days when data were collected or the animal was trained on the behavioral paradigm. On these days, the animals were allowed unlimited access to fluid through their performance in the behavioral paradigm. Here, the animals received fluid rewards for every correctly performed trial. Throughout the study, the animals’ psychological and veterinary wellbeing was monitored by the veterinarians, the animal facility staff, and the lab’s scientists, all specialized on working with nonhuman primates.

Both of the animals used in the study are currently in other studies in our laboratory.

### General

We trained two male rhesus monkeys (*Macaca mulatta*), monkey H and monkey E, to perform a demanding visuospatial-attention task along with a saccade. Each monkey was implanted with a titanium head holder and a recording chamber located above the parietal cortex (based on a MRI scan) to allow MT recordings. All surgical procedures were approved by the district government of Lower Saxony, Germany, and were conducted under general anesthesia using standard techniques. The experiments were performed in a dimly lit room, and the monkey viewed a CRT monitor (76 Hz) while sitting in a custom-made primate chair during the experiment (see S1 Text for detailed Methods). All aspects of the experiment were controlled by custom software running on an Apple Macintosh computer. The eye position was monitored by an EyeLink 1000 (SR Research, Canada) system at 1,000 Hz. Neuronal activity was recorded extracellularly with a 5-channel micro drive system (Mini Matrix, Thomas Recording, Giessen, Germany) and processed using the Plexon data acquisition system (Plexon Inc., Dallas, TX, United States). Only data from well-isolated neurons are reported here. MT was identified by referencing the recordings to the structural MRI and by the physiological properties of the recorded neurons.

### Behavioral Tasks and Stimuli

Once a neuron was isolated, we mapped its RF location and determined the neurons’ preferred direction and speed while the monkeys performed a fixation task. We then switched to the main experiment (Fig 1), in which each trial was composed of one of four tasks (three experimental tasks and one control task, chosen in a pseudo-randomly interleaved manner). In all four tasks, the monkeys initiated the trial by holding a metal bar and foveating a black fixation point. In the control task (the “simple-saccade” task, 11.1% of trials), the monkeys had to maintain fixation until a saccade target (identical to the fixation point) appeared and the fixation point disappeared (see S1 Text for details). The monkeys had to make a saccade to the saccade target and maintain fixation there until the end of the trial to obtain a reward. In the three experimental tasks, in addition to potentially making a saccade as in the simple-saccade task, the monkeys had to attend to one of two moving RDPs (both moving in the same direction, which was either the neuron’s preferred or antipreferred direction) and respond to a brief (132 ms) direction change in this target by releasing the bar, but ignore similar changes in the other RDP (the distractor). The target stimulus was cued by a stationary RDP that appeared at its location for 263 ms. The target and distractor stimuli were always equidistant from the fixation point and saccade target and were always mirrored with respect to the saccade target (Fig 1), so that for horizontal saccades, they appeared in the upper and lower hemifield (and the left or right hemifield, if the RF was located in the left [monkey H] hemifield or right [monkey E], respectively). The cue appeared equally often in the postsaccadic RF (attend-in condition) or opposite to it (attend-out condition). In addition, during the trial, if the fixation point jumped to a new location (as in the simple-saccade task), the monkeys had to refixate the fixation point while continuing to attend to the cued target. In the first of the three experimental tasks (the “continuous-stimulus task,” 22.2% of trials), the fixation point jumped to its new location 671 ms after RDP onset. The direction change in the target RDP could occur between 974 and 1,895 ms after the fixation point jumped. The second experimental task (the “interrupted-stimulus task,” 44.4% of trials) was similar to the continuous-stimulus task, but the target and distractor RDPs disappeared 105 ms after the fixation point jumped and, therefore, no stimulus ever appeared in the neurons’ RF after the saccade (or before the saccade). The monkeys had to simply make a saccade to the new fixation point location and maintain fixation until the end of the trial to obtain a reward; the few trials with saccades that started before the disappearance of the stimulus were discarded. The third experimental task (the “fixation task,” 22.2% of trials) was also similar to the continuous-stimulus task except that the fixation point never jumped, and the direction change in the target RDP occurred 789 to 1,842 ms after RDP onset. This task was included to make sure the monkeys paid attention to the target even during the time when they made a saccade in the other two experimental tasks, and was not analyzed further for this study. Distractor changes occurred on about 37.5% of trials (in the continuous-stimulus and fixation tasks) and never more than once on each trial. The timing of distractor changes overlapped that of target changes, with the additional requirement that any distractor change occurred at least 500 ms before the target change on each trial. This separation ensured that the monkeys’ rare responses to the distractor change could be easily identified and distinguished from their responses to the target change. In all the tasks, the background was always grey, and the fixation point and RDPs, including the stationary cue, were black. Our use of black stimuli addresses concerns regarding the persistence of white visual stimuli on black backgrounds after their stipulated disappearance from a CRT monitor.

### Data Analysis

We detected saccades using a velocity threshold criterion that was validated by visual inspection. We included data from all neurons that showed a significantly greater postsaccadic response to at least one of the two directions in the continuous-stimulus task (compared to the simple saccade task in which there is no stimulus in the RF, i.e., they were visually responsive to the RDP) as well as a significant difference between the responses to the two RDP directions in the continuous-stimulus task (i.e., they showed direction tuning). Additionally, we excluded neurons in which the onset of the RDP at the (future) postsaccadic RF location elicited a statistically significant response from the neuron. Only correctly completed trials were analyzed. PSTHs (Figs 2–4) were calculated using partially overlapping bins (50 ms width, stepped every 10 ms). For the interrupted-stimulus task (Figs 3–5), we used a time window from 0 to 350 ms after saccade offset, as a compromise duration that was long enough to make statistically meaningful statements about the effects we observed, and yet not so long that the monkeys would have ample time to withdraw attention from the attended spatial location after realizing that the attended stimulus had disappeared. In addition, 350 ms is roughly equal to a typical intersaccadic interval. For the continuous-stimulus task (Fig 2), our goal was to merely confirm that we found the attentional effects expected from MT in our dataset. For a precise estimate, we chose a time window of 0 to 500 ms. This choice is not critical, and using a time window of 0 to 350 ms would not affect our conclusions (though it would provide a less precise estimate). The modulation index was defined as the difference in the firing rates for the two conditions divided by their sum. A direction-tuning index was similarly defined as the difference in firing rates for preferred and antipreferred directions divided by their sum (Fig 5). We report medians and use *p*-values from Wilcoxon signed-rank tests throughout.

## Supporting Information

S1 Fig The memory trace does not start earlier than the sensory response. The memory trace, plotted as the difference between the response in the attend-in condition of the interrupted-stimulus task and the response in the simple-stimulus task (mean difference across neurons and SEM—red trace), arises at the same time or later than the sensory response, plotted as the difference between the response in the continuous-stimulus task with the preferred direction and the response in the simple-stimulus task (mean difference across neurons and SEM—blue trace). The contribution of predictive remapping to the timing of the steep rise of the sensory response toward its peak would be minimal, and the memory trace does not appear to lead the sensory response anywhere along this steep rise. In order to facilitate comparison, both traces were normalized by subtracting the mean value of the trace from -300 to 0 ms and then dividing by the maximum value. Data for monkey H (A) and monkey E (B). Other conventions as in Fig 2. Data in Supporting Information (S6 Data).
(PDF)

S2 Fig Attentional effects in the continuous task manifest at or after saccade offset. The attentional effect in the continuous-stimulus task for the preferred direction, plotted as the difference between the responses in the attend-in (Fig 2, blue curve) and attend-out (Fig 2, red curve) conditions (mean and 95% confidence bands), rises above zero only after saccade offset. Data for monkey H (A) and monkey E (B). Other conventions as in Fig 2. Data in Supporting Information (S7 Data).
(PDF)

S1 Data The data presented in Fig 2 are tabulated in separate text files contained in the zipped folder. (ZIP)

S2 Data The data presented in Fig 3 are tabulated in separate text files contained in the zipped folder. (ZIP)

S3 Data The data presented in Fig 4 are tabulated in separate text files contained in the zipped folder. (ZIP)

S4 Data The data presented in Fig 5 (and in the associated portion of the Results section) are tabulated in separate text files contained in the zipped folder. (ZIP)

S5 Data The data presented in Fig 6 are tabulated in separate text files contained in the zipped folder. (ZIP)

S6 Data The data presented in S1 Fig are tabulated in separate text files contained in the zipped folder. (ZIP)

S7 Data The data presented in S2 Fig are tabulated in separate text files contained in the zipped folder. (ZIP)

S1 Text Detailed Materials and Methods. (PDF)

## Abbreviations

FEF: frontal eye field
LIP: lateral intraparietal area
MST: medial superior temporal area
MT: middle temporal area
PSTH: peristimulus time histogram
RDP: random dot pattern
RF: receptive field
SC: superior colliculus
