# Supplementary figures and images for: An Attention-Sensitive Memory Trace in Macaque MT Following Saccadic Eye Movements
Source: PLoS Biol. 2016 Feb 22;14(2):e1002390. doi: 10.1371/journal.pbio.1002390 (PMC4764326; doi:10.1371/journal.pbio.1002390)

- Sensory response
- Memory trace

### Monkey H

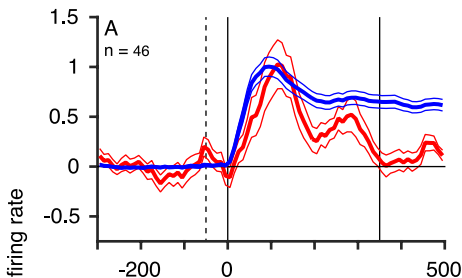

### Monkey E

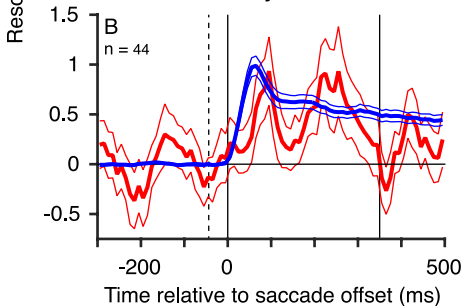

Supplement: S1 Fig — The memory trace, plotted as the difference between the response in the attend-in condition of the interrupted-stimulus task and the response in the simple-stimulus task (mean difference across neurons and SEM—red trace), arises at the same time or later than the sensory response, plotted as the difference between the response in the continuous-stimulus task with the preferred direction and the response in the simple-stimulus task (mean difference across neurons and SEM—blue trace). The contribution of predictive remapping to the timing of the steep rise of the sensory response toward its peak would be minimal, and the memory trace does not appear to lead the sensory response anywhere along this steep rise. In order to facilitate comparison, both traces were normalized by subtracting the mean value of the trace from -300 to 0 ms and then dividing by the maximum value. Data for monkey H (A) and monkey E (B). Other conventions as in Fig 2. Data in Supporting Information (S6 Data). (PDF) [file pbio.1002390.s001.pdf]

## Monkey H

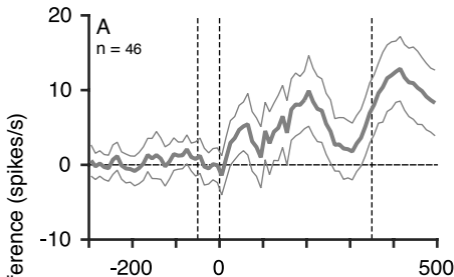

## Monkey E

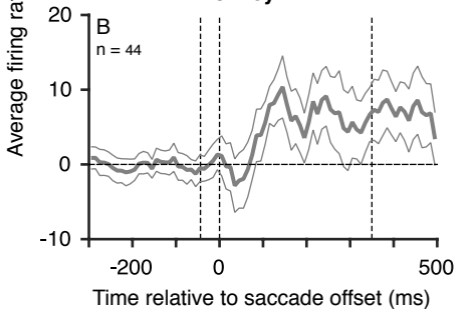

Supplement: S2 Fig — The attentional effect in the continuous-stimulus task for the preferred direction, plotted as the difference between the responses in the attend-in (Fig 2, blue curve) and attend-out (Fig 2, red curve) conditions (mean and 95% confidence bands), rises above zero only after saccade offset. Data for monkey H (A) and monkey E (B). Other conventions as in Fig 2. Data in Supporting Information (S7 Data). (PDF) [file pbio.1002390.s002.pdf]
