# Supplementary material for: An Attention-Sensitive Memory Trace in Macaque MT Following Saccadic Eye Movements
Source: PLoS Biol. 2016 Feb 22;14(2):e1002390. doi: 10.1371/journal.pbio.1002390 (PMC4764326; doi:10.1371/journal.pbio.1002390)
Supplement: S1 Text — (PDF) [file pbio.1002390.s010.pdf]

Supporting information

Methods

This is an expanded version of the Methods section in the main text. We trained two male rhesus monkeys (Macaca mulatta, 7–11 kg), monkey H and monkey E, to perform a demanding visuospatial-attention task along with a saccade. Each monkey was implanted with a titanium head holder to minimize head movements during the experiment. One recording chamber was also implanted in each monkey above the left (monkey E) or the right (monkey H) parietal cortex to allow access to MT, with implantation locations chosen based on a preceding MRI scan. All procedures were approved by the district government of Lower Saxony, Germany, and all surgeries were conducted under general anesthesia using standard techniques.

The experiments were performed in a dimly-lit room with the only source of light being the display monitor. A CRT monitor (Sony Trinitron GDM-FW900) at a distance of 57 cm from the monkey was used to display the visual stimulus at a refresh rate of 76 Hz and a spatial resolution of 40 pixels/degree. The monkey sat in a custom-made primate chair during the experiment. Stimulus presentation, reward delivery, electrophysiological and behavioral data collection was controlled by custom software and run on an Apple Macintosh computer. All stimulus onsets and durations were specified in terms of number of frames (CRT monitor refreshes), and the times reported here in millisecond units are correct to within 13 ms (the duration of one frame), given the vertical scan-rate properties of the CRT monitor. The animals received a fluid reward immediately following each correct trial. The eye-position was monitored by an EyeLink 1000 (SR Research, Canada) system at 1000 Hz. Neuronal activity was recorded extracellularly with a 5-channel micro drive system (Mini Matrix, Thomas Recording, Giessen, Germany) and processed using the Plexon data acquisition system (Plexon Inc., Dallas, TX). Only data from well-isolated neurons were used for the analysis. MT was identified by referencing the recordings to the structural MRI and by the physiological properties of the recorded neurons: most

neurons were direction-tuned, the average diameter of the receptive fields (RFs) was approximately equal to the RF eccentricity and there was a predictable progression of RF centers at different locations along the superior temporal sulcus.

Behavioral tasks and stimuli

Once a neuron was isolated and while the monkey performed a fixation task, we located the RF by moving a stationary circular random dot pattern (RDP) across the screen using a mouse. During this task, the monkey had to maintain fixation on a fixation point and respond to a brief luminance change at the fixation point. We then determined the neuron’s preferred direction and speed, again while the monkey performed a fixation task (usually at the location of the saccade target in the main task), by presenting a RDP with moving dots within a circular aperture in the RF, changing the direction and speed every 250 ms picked from a set of 3 possible speeds (4, 8 or 16 degrees per second) and 12 possible directions (evenly separated by 30 degrees around a circle). The preferred and anti-preferred directions, and the preferred speed were used in the main experiment. Occasionally, we recorded simultaneously from two or more neurons with overlapping RFs when their preferred directions also overlapped or were opposite to each other.

After identifying the RF location and preferred direction, we switched to the main experiment. In the main experiment (Figure 1), each trial was composed of one of four tasks (three experimental tasks and one control task, chosen in a pseudo-randomly interleaved manner). For all four tasks, the monkeys initiated the trial by holding a lever and foveating a black fixation point. In the control task (the “simple-saccade” task), performed on 11.1 % of trials, a saccade target, identical to the fixation point, appeared between 10 and 20 degrees eccentrically (value fixed for each neuron, and either 15 or 20 degrees in most cases),1382 ms after the monkey initiated fixation. The saccade target then stayed on for 1368 ms (the end of the trial). Saccades were always either horizontal or vertical. In all tasks, there was a

one-frame (13 ms) overlap between the fixation point and the saccade target, so that the fixation point disappeared one frame after the saccade target appeared: perceptually, the fixation point appeared to jump from its original location to the saccade target. Once the fixation point jumped, the monkey had to make a saccade to the new location of the fixation point within 263 ms and maintain fixation until the end of the trial in order to obtain a reward for correct performance. In the three experimental tasks, after 118 ms of fixation, a stationary RDP cue within a circular aperture (of the same size as the target), which indicated the location of the future target of attention, was presented for 263 ms either in the neurons' post-saccadic RF (attend-in condition) or opposite to it (attend-out condition) equally often. After an additional delay of 329 ms following cue offset, two moving RDPs (2 degrees in radius, all dots moving in the same direction of motion and within stationary circular apertures) were presented on the screen, with both moving equally often in the neurons' preferred or anti-preferred direction. One of them, the target was located at the previously cued location, while the other, the distractor, was located opposite to it (i.e. reflected across the horizontal or vertical meridian, see Figure 1). The monkeys' task was to respond to a brief (132 ms) direction change in the target by releasing the lever (within 600 ms of the change), but ignore similar changes in the distractor. In addition, during the trial, if the fixation point jumped to a new location (as in the "simple-saccade" task), the monkeys had to refixate the fixation point while continuing to attend to the cued target. In the first of the three experimental tasks (the "continuous-stimulus task", 22.2 % of trials), the fixation point jumped to its new location 671 ms after RDP onset. The direction change in the target RDP could occur between 974 ms to 1895 ms after the fixation point jumped. The second experimental task (the "interrupted-stimulus task", 44.4 % of trials) was similar to the continuous-stimulus task, and the fixation point jumped at the same time as in the continuous-stimulus task, but the target and distractor RDPs disappeared 105 ms after the fixation point jumped. Therefore, on these trials, no stimulus ever appeared in the neurons' RF after the saccade (or before the saccade). The monkeys had to simply make a saccade to the new fixation point location and maintain fixation until the end of the trial to obtain a reward. The

few trials with saccades that started before the disappearance of the stimulus were discarded. The third experimental task (the “fixation task”, 22.2 % of trials) was also similar to the continuous-stimulus task, except that the fixation point never jumped, and the direction-change in the target RDP occurred 789 ms to 1842 ms after RDP onset. This task was included to make sure the monkeys paid attention to the target even during the time when they made a saccade in the other two experimental tasks, and was not analyzed further for this study.

In all the tasks, the background was always grey with a luminance of 14.2 cd/m^2, and the fixation point and RDPs including the stationary cue were black with the luminance of 0.68 cd/m^2. Our use of black stimuli minimizes the concerns that arise when white stimuli are used on a black background regarding the persistence of visual stimuli on the display monitor after their stipulated disappearance. Individual RDP dot size was 0.1° x 0.1°, and the dot density was 10 dots/deg^2. Monkeys had to maintain fixation within a circular window of 2 degrees radius around the fixation point, except for a period of 263 ms after the fixation point jumped to give them time to make the saccade. The saccade direction was set according to the position of the RF: for example, if the RF center was directly above or below the fixation point, we used a horizontal saccade, while if the RF center was directly to the left or right of the fixation point, we used a vertical saccade. If the RF center was offset both vertically and horizontally from the fixation point, the choice was no longer critical, but we usually used a horizontal saccade.

Data analysis

We only included correctly performed trials in our analysis. After excluding fixation breaks, both monkeys performed the tasks correctly on over 94.6 % of trials. Data analysis was performed using custom software in MATLAB (MATLAB Inc, Natick, MA). We included data from all neurons that showed a

significantly greater post-saccadic response to at least one of the two directions in the continuous-stimulus task (compared to the simple saccade task where there is no stimulus in the RF, i.e. they were visually responsive to the RDP) as well as a significant difference between the responses to the two RDP directions in the continuous-stimulus task (i.e. they showed direction tuning). Additionally, we excluded neurons where the onset of the RDP at the (future) post-saccadic RF location elicited a statistically significant response from the neuron. Peri-stimulus time histograms (PSTHs: Figures 2-4) were calculated using partially overlapping bins (50 ms width, stepped every 10 ms): the mean activity for each neuron across trials was first calculated and then these mean PSTHs for individual neurons were averaged across neurons to obtain the displayed PSTHs. For the differences between conditions (say A and B), we report the effects using a modulation index, which is defined in the usual manner for each neuron as the difference in the firing-rates for the two conditions divided by their sum. A direction-tuning index was similarly defined as the difference in firing-rates for preferred and anti-preferred directions divided by their sum (Figure 5). We report the average modulation and direction-tuning indices using the median value after converting it back into a percentage. For the analyses of the memory trace in Figures 3 and 4, because we did not find a difference between the response in the interrupted-stimulus task based on whether the RDP was moving in the preferred or anti-preferred direction before the saccade (Figure 5), we pooled the trials irrespective of RDP direction and only separated the trials into those where the monkey was cued to attend to the post-saccadic RF location (attend-in condition) and those where the monkey was cued to attend outside it (attend-out condition).

We detected saccades using a standard velocity-threshold algorithm: onset (and offset) times were determined by when the eye velocity exceeded (and then dropped below) 30 degrees per second. This threshold value was set to lie clearly above the peak excursions of the baseline noise in the eye-velocity traces, and the algorithm was validated by visual inspection for each monkey. In addition to the large saccade from the fixation point to the saccade target (to which we aligned our data for our analyses), both

monkeys also made small-amplitude eye-movements within the fixation window while fixating: these eye-movements could be observed to induce a short-duration transient response lasting less than 100 ms when there was a motion stimulus in the RF [as has been reported earlier: 1]. For the main analyses of interest in this paper, there was no stimulus in the RF after the saccade and no transient response was detected following the microsaccade. Even so, we re-examined our results and conclusions after discarding spikes elicited within 100 ms after any small eye movement occurring after the large refixation saccade, within the time-windows of interest. This correction was made both for the PSTHs and for the statistical comparisons based on spike-counts. We verified that our results remain similar and that our conclusions remain robust when such a correction for small eye-movements is made.

1. Herrington TM, Masse NY, Hachmeh KJ, Smith JE, Assad JA, Cook EP. The effect of microsaccades on the correlation between neural activity and behavior in middle temporal, ventral intraparietal, and lateral intraparietal areas. The Journal of neuroscience : the official journal of the Society for Neuroscience. 2009;29(18):5793-805.
